# Life threatening shrimp allergy cross reacting with mite allergy : a case report

**DOI:** 10.1186/1710-1492-10-S1-A9

**Published:** 2014-03-03

**Authors:** Farag I Farag-Mahmod, Waheed I Hessam

**Affiliations:** 1 Suez Canal University Hospital, Faculty of Medicine, Allergy & Immunology Unit, Egypt; 2 Omega laboratories Ltd, Montreal, Quebec, Canada

## Background

Food allergy is apparently increasing and it is estimated that 3–4% of adults have food allergy. Shrimp is one of the most common causatives in seafood allergy. Patients with shrimp allergy may exhibit life threatening anaphylactic reactions. Tropomyosin is a known important allergen in shrimp. The IgE-binding epitope in shrimp tropomyosin, cross-reacts with other allergenic invertebrate tropomyosins in house dust mites (Der p 10, Der f 10) and cockroaches (Per a 7).

## Objective

this study was undertaken to evaluate the results of immunotherapy to Mites upon allergic reactions to shrimp allergen in a 40 years old female suffering from combined allergy to mites and shrimp. The patient had a 10 years history of severe allergic reactions after eating shrimp; for which she was hospitalized several times to receive intravenous steroids and antihistamines. Despite avoiding consumption of shrimp for several years she continued to have allergic rhinitis symptoms. The last episode of allergic reactions to shrimp occurred while cooking shrimp not eating it.

## Materials and methods

Allergy skin prick testing against a panel of 30 common allergens including Mites and shrimp ( Omega, Montreal, Canada) revealed a strong positive wheal and erythema reaction to both shrimp and D. farinae (13mm, 9 mm for wheal and 2.5 cm, 2.3 cm for erythema respectively). Allergen specific serum IgE testing also revealed elevated serum specific IgE to both allergens. The patient started subcutaneous allergen specific immunotherapy (ASIT) against D. farinae using Omega labs allergy shots. Six months after ASIT there was a significant reduction in the size of the wheal and erythema and also significant decrease of serum specific IgE values for both allergens.

## Conclusion

Subcutaneous immunotherapy against mites may desensitize patients against shrimp allergy.

